# The Role of Glycemic Control in Inflammation Markers and Clinical Outcomes in Type 2 Diabetes Patients with Severe COVID-19

**DOI:** 10.3390/biomedicines13040886

**Published:** 2025-04-06

**Authors:** Lavinia Craciun, Flavia Ignuta, Uma Shailendri Rayudu, Maliha Afra, Ovidiu Rosca, Adrian Vlad, Oana Aburel, Dana Emilia Velimirovici

**Affiliations:** 1 Department of Anatomy and Embryology, “Victor Babes” University of Medicine and Pharmacy Timisoara, Eftimie Murgu Square 2, 300041 Timisoara, Romania; craciun.lavinia@umft.ro; 2 Doctoral School, “Victor Babes” University of Medicine and Pharmacy Timisoara, Eftimie Murgu Square 2, 300041 Timisoara, Romania; 3 Department of Infectious Disease, “Victor Babes” University of Medicine and Pharmacy Timisoara, Eftimie Murgu Square 2, 300041 Timisoara, Romania; ovidiu.rosca@umft.ro; 4 Centre for Molecular Research in Nephrology and Vascular Disease, Faculty of Medicine, “Victor Babes” University of Medicine and Pharmacy Timisoara, Eftimie Murgu Square 2, 300041 Timisoara, Romania; vlad.adrian@umft.ro; 5 Medical School, Gitam Institute of Medical Sciences and Research, Visakhapatnam, Andhra Pradesh 530045, India; uma.shailendri@gmail.com; 6 Medical School, Deccan College of Medical Sciences, Hyderabad, Telangana 500058, India; malihaafra04@gmail.com; 7 Department of Internal Medicine II, Division of Diabetes, Nutrition and Metabolic Diseases, “Victor Babes” University of Medicine and Pharmacy Timisoara, Eftimie Murgu Square 2, 300041 Timisoara, Romania; 8 Department III Functional Sciences—Pathophysiology, “Victor Babes” University of Medicine and Pharmacy Timisoara, Eftimie Murgu Square 2, 300041 Timisoara, Romania; oanaduicu@umft.ro; 9 Center for Translational Research and Systems Medicine, “Victor Babes” University of Medicine and Pharmacy Timisoara, Eftimie Murgu Square 2, 300041 Timisoara, Romania; 10 Department VI Cardiology, Internal Medicine and Ambulatory Care, Prevention and Cardiovascular Recovery, “Victor Babes” University of Medicine and Pharmacy Timisoara, Eftimie Murgu Square 2, 300041 Timisoara, Romania; dana.velimirovici@umft.ro

**Keywords:** COVID-19, diabetes mellitus type 2, glycemic control, inflammation mediators, severity of illness index

## Abstract

**Background and Objectives:** Patients with type 2 diabetes mellitus (T2DM) are at a heightened risk of adverse outcomes from Coronavirus Disease 2019 (COVID-19). However, the influence of glycemic control on systemic inflammation and clinical severity remains incompletely understood. This study aimed to compare inflammatory markers, composite severity scores, and clinical outcomes between T2DM patients with COVID-19 who had well-controlled diabetes (hemoglobin A1c < 7%) versus those with poorly controlled diabetes (hemoglobin A1c ≥ 7%). **Methods:** We retrospectively reviewed 181 adult patients with T2DM and severe COVID-19 admitted to a tertiary hospital between January 2022 and December 2023. Patients were divided into two groups: well-controlled (*n* = 117) and poorly controlled (*n* = 64) T2DM. Baseline demographics, comorbidities, and laboratory parameters (C-reactive protein, interleukin-6, ferritin, neutrophil and lymphocyte counts, platelets, and calculated indices such as the neutrophil-to-lymphocyte ratio [NLR] and systemic immune-inflammation index [SII]) were collected. Composite severity scores (APACHE II, CURB-65, and NEWS) and clinical outcomes (ICU admission, mechanical ventilation, mortality, and length of stay) were compared. Statistical tests used included Student’s *t*-test or the Mann–Whitney U for continuous variables and chi-square for categorical variables, with *p* < 0.05 deemed significant. **Results:** The two groups did not differ significantly in age or duration of diabetes (*p* = 0.40 and *p* = 0.75, respectively). Patients with poorly controlled T2DM exhibited higher inflammatory markers (mean CRP of 93.4 mg/L vs. 78.6 mg/L, *p* = 0.002; IL-6 of 64.2 pg/mL vs. 52.8 pg/mL, *p* = 0.004) and elevated severity scores (APACHE II of 16.8 vs. 14.1, *p* = 0.001). Poor glycemic control was associated with higher ICU admissions (39.1% vs. 22.2%, *p* = 0.02) and mortality (14.1% vs. 6.0%, *p* = 0.05). **Conclusions:** In T2DM patients hospitalized with COVID-19, poor glycemic control correlates with heightened inflammatory responses, worse composite severity scores, and increased rates of unfavorable outcomes. These findings underscore the necessity of stringent glucose management to mitigate inflammation and improve prognoses in this vulnerable patient population.

## 1. Introduction

Coronavirus Disease 2019 (COVID-19), caused by SARS-CoV-2, continues to challenge global healthcare systems, although the COVID-19 pandemic ended in 2023 according to the World Health Organization [1,2]. Despite significant progress in vaccination and antiviral treatments, select populations remain at higher risk for severe forms of the illness [3,4]. Among these groups, patients with type 2 diabetes mellitus (T2DM) stand out due to their predisposition to adverse events and increased healthcare utilization [5,6,7].

Hyperglycemia has been identified as an aggravating factor in infections, primarily by impairing immune cell function and promoting inflammatory pathways [8,9]. In COVID-19, poor glycemic control has been associated with elevated risks of hospitalization, mechanical ventilation, and death [10,11]. Nevertheless, the precise links between glycemic status, inflammatory markers, and clinical severity scores have not been thoroughly elucidated [12,13].

Several clinical severity tools—such as the Acute Physiology and Chronic Health Evaluation II (APACHE II), the CURB-65 pneumonia severity scale, and the National Early Warning Score (NEWS)—have been widely used to evaluate prognosis in acutely ill patients [14,15,16]. Concurrently, inflammatory indices like the neutrophil-to-lymphocyte ratio (NLR) and systemic immune-inflammation index (SII) have emerged as reliable indicators of systemic inflammation, correlating with outcomes in COVID-19 [17,18]. Chronic hyperglycemia in T2DM can exacerbate low-grade systemic inflammation, perpetuating a cycle of immune dysfunction [19,20]. When compounded by an acute inflammatory stressor like COVID-19, patients with poorly controlled diabetes may experience disproportionately severe disease courses [21,22].

Early identification of high-risk individuals is essential to improve resource allocation, optimize glycemic control measures, and potentially adapt immunomodulatory or anti-inflammatory treatments. Therefore, the present study investigates the impact of glycemic control on inflammation, severity indices, and outcomes among hospitalized COVID-19 patients with T2DM. By comparing patients with well-controlled and poorly controlled diabetes based on hemoglobin A1c levels (HbA1c < 7% vs. HbA1c ≥ 7%) [22,23], we aim to contribute clinically relevant evidence that can shape intervention protocols and reduce morbidity and mortality in this high-risk population.

## 2. Materials and Methods

### 2.1. Study Design and Population

This retrospective cohort study was conducted at a tertiary-care academic hospital in Timisoara, Romania, at the Victor Babes Hospital for Infectious Disease and Pulmonology. Eligible patients were those admitted with reverse-transcriptase polymerase chain reaction (RT-PCR)-confirmed COVID-19 and a documented history of type 2 diabetes mellitus. The primary measure was glycemic control, defined by the most recent hemoglobin A1c measurement during the three months preceding hospital admission. Medical charts were thoroughly reviewed to identify patients who met the inclusion criteria. For each enrolled participant, data on demographic characteristics, comorbid conditions, and laboratory test results were retrieved. The research protocol was approved by the institutional review board, and the requirement for informed consent was waived due to the retrospective nature of the study.

### 2.2. Patient Selection and Grouping

Adult patients (≥18 years) with T2DM were included if they presented with an acute COVID-19 infection requiring inpatient care for severe infection. To form comparison groups, patients were classified into either the “well-controlled T2DM” group (HbA1c < 7.0%) or the “poorly controlled T2DM” group (HbA1c ≥ 7.0%) [24]. We excluded individuals with incomplete medical records regarding HbA1c or those with a known history of type 1 diabetes. We also excluded patients with immunodeficiency syndromes unrelated to diabetes, advanced malignancy with end-of-life care, or severe chronic liver disease, as these conditions could confound inflammatory marker interpretations. The final analysis comprised 181 patients: 117 in the well-controlled group and 64 in the poorly controlled group. A third group of patients with COVID-19 without diabetes was included as the control.

In this study, severe COVID-19 was defined according to established clinical and physiological parameters, including at least one of the following: respiratory rate ≥ 30 breaths per minute, oxygen saturation (SpO₂) < 94% on room air, PaO₂/FiO₂ ratio < 300 mmHg, or the presence of lung infiltrates affecting more than 50% of the lung fields. Type 2 diabetes mellitus (T2DM) was diagnosed based on standard criteria, which included a history of non-insulin-dependent hyperglycemia, the requirement of oral hypoglycemic agents or insulin for glycemic control, or documentation of an HbA1c ≥ 6.5% (48 mmol/mol) as per American Diabetes Association guidelines.

### 2.3. Data Collection and Variables

Baseline demographic and clinical data included age, sex, body mass index (BMI), duration of diabetes, and the presence of comorbidities such as hypertension and chronic kidney disease. Key laboratory parameters were recorded, such as complete blood count, C-reactive protein (CRP), interleukin-6 (IL-6), ferritin, and serum albumin. From these measures, we calculated the NLR (neutrophil count/lymphocyte count) and the SII ([platelet count × neutrophil count]/lymphocyte count). Clinical severity was assessed using validated scores from APACHE II, CURB-65, and NEWS, each calculated within the first 24 h of admission. Primary outcomes included ICU admission, the need for mechanical ventilation, and in-hospital mortality. Secondary outcomes included length of hospital stay and correlation analyses between inflammatory markers and severity scores.

### 2.4. Statistical Analysis

All statistical analyses were performed using SPSS version 27 (IBM Corp., Armonk, NY, USA). Continuous variables were summarized as mean ± standard deviation and compared using Student’s *t*-test if normally distributed, or the Mann–Whitney U test if distribution was non-normal. Categorical variables were reported as frequencies (percentages) and compared using the chi-square test or Fisher’s exact test, as appropriate. Correlations between laboratory markers and clinical severity scores were explored using the Pearson or Spearman correlation coefficients, contingent upon normality assumptions. Logistic regression was utilized to identify independent predictors of severe outcomes (ICU admission, mechanical ventilation, or death). A *p*-value below 0.05 was considered statistically significant throughout all analyses.

## 3. Results

### Demographics

Table 1 outlines the baseline characteristics of the two study groups: T2DM patients with COVID-19 who have either well-controlled diabetes (HbA1c < 7.0%) or poorly controlled diabetes (HbA1c ≥ 7.0%). The mean age was comparable between the groups (62.7 years vs. 63.9 years, *p* = 0.40), suggesting that age is unlikely to confound subsequent outcome comparisons. The proportion of male patients was also similar (54.7% vs. 59.4%, *p* = 0.50). Critically, both groups had nearly the same average duration of diabetes (10.2 vs. 10.4 years, *p* = 0.75), indicating that any observed differences in inflammatory markers or clinical outcomes are not simply attributable to longer-standing T2DM in one group. One significant finding was the higher mean BMI in the poorly controlled diabetes group compared to the well-controlled group (30.2 kg/m^2^ vs. 28.4 kg/m^2^, *p* = 0.006). Although both means fall into the overweight/obesity range, this difference might reflect distinct metabolic profiles and could partially contribute to an increased inflammatory burden. Hypertension and chronic kidney disease (CKD) were prevalent in both cohorts (*p* > 0.05 for both), aligning with the known cardiometabolic risk profiles often present in T2DM.

Table 2 presents a comparison of key inflammatory and laboratory markers between the two glycemic control groups. Notably, patients with poorly controlled T2DM had significantly higher average CRP levels (93.4 mg/L vs. 78.6 mg/L, *p* = 0.002) and IL-6 concentrations (64.2 pg/mL vs. 52.8 pg/mL, *p* = 0.004), suggesting a more pronounced systemic inflammatory response. Ferritin, another acute-phase reactant, was also elevated in the poorly controlled group (524.7 µg/L vs. 449.2 µg/L, *p* = 0.01), underscoring the potential for heightened immune activation. Regarding white blood cell parameters, lymphocyte counts were lower in the poorly controlled cohort (1.0 vs. 1.2 × 10^9^/L, *p* = 0.003), which often signals impaired immunologic resilience. The neutrophil-to-lymphocyte ratio (NLR), a validated marker of systemic inflammation and prognostic indicator in COVID-19, was substantially higher in the poorly controlled group (7.2 vs. 5.7, *p* < 0.001). Similarly, the systemic immune-inflammation index (SII) was markedly elevated (1344.7 × 10^3^ vs. 1039.3 × 10^3^, *p* < 0.001).

Table 3 shows three widely used clinical severity scores—APACHE II, CURB-65, and NEWS—measured at hospital admission in both study groups. Patients with poorly controlled diabetes demonstrated higher mean APACHE II scores (16.8 vs. 14.1, *p* = 0.001), indicating a more precarious physiologic status upon presentation. Since APACHE II incorporates variables such as vital signs, serum biochemistry, and chronic health status, the elevated score in the poorly controlled cohort may reflect the cumulative burden of hyperglycemia and inflammation. CURB-65, a pneumonia severity score that evaluates confusion, urea, respiratory rate, blood pressure, and age, was also significantly higher (2.5 vs. 2.1, *p* = 0.03) in patients with poor glycemic control. This difference suggests that they may be at a greater risk of severe respiratory compromise. In parallel, NEWS, which integrates respiratory rate, oxygen saturation, systolic blood pressure, pulse, level of consciousness, and temperature, was higher in the poorly controlled group (7.7 vs. 6.6, *p* = 0.007).

Table 4 focuses on key clinical outcomes, showing that glycemic control appears to significantly affect patient trajectories. ICU admission rates were notably higher in the poorly controlled group than in the well-controlled cohort (39.1% vs. 22.2%, *p* = 0.02). This underscores that chronic hyperglycemia may predispose patients to more severe presentations requiring intensive supportive measures. While the difference in the mechanical ventilation rate did not reach conventional significance (18.8% vs. 10.3%, *p* = 0.10), the numeric gap aligns with the overall trend of increased severity in poorly controlled T2DM patients. Mortality was more than double in the poorly controlled group (14.1% vs. 6.0%, *p* = 0.05), approaching statistical significance and highlighting the potential lethality linked to suboptimal glycemic status in the context of COVID-19. The length of hospital stay also differed, with poorly controlled patients averaging 14.4 days compared to 12.7 days for those with better glycemic regulation (*p* = 0.01).

Table 5 delves into selected subgroup analyses based on age (≥65 years) and obesity (BMI ≥ 30 kg/m^2^), two well-documented risk factors for poor COVID-19 outcomes. The proportion of older patients was comparable between groups (46.2% vs. 48.4%, *p* = 0.77), indicating similar age distributions. Interestingly, among these older patients, ICU admission rates were higher in the poorly controlled cohort (41.9% vs. 29.6%), although the difference did not reach statistical significance (*p* = 0.23). Still, the higher ICU rate is consistent with a pattern of more severe illness in poorly controlled diabetes, especially in advanced age. In contrast, obesity was significantly more prevalent in the poorly controlled group compared to the well-controlled group (48.4% vs. 30.8%, *p* = 0.02). This finding aligns with the higher mean BMI observed earlier (Table 1). Although the difference in ICU admissions for obese patients also trended higher in the poorly controlled group (38.7% vs. 25.0%, *p* = 0.24), the relatively small subgroup sample may have limited the statistical power to detect a difference.

Table 6 illustrates the correlations between various inflammatory indicators, clinical severity scores, and ICU admission, a key marker of severe disease. The correlation coefficient (r) values range from moderate to strong. APACHE II shows the strongest association (r = 0.51, *p* < 0.001) with ICU admission, consistent with its comprehensive physiological assessment framework. IL-6, a cytokine intimately involved in the inflammatory cascade, also exhibits a notable correlation (r = 0.45, *p* < 0.001), underscoring its prognostic relevance in COVID-19. Markers like CRP (r = 0.41, *p* < 0.001) and the neutrophil-to-lymphocyte ratio (NLR) (r = 0.38, *p* < 0.001) further reflect the systemic inflammatory burden that appears to drive or coincide with disease severity. The systemic immune-inflammation index (SII) correlates moderately with ICU admission (r = 0.33, *p* = 0.001), highlighting that platelet, neutrophil, and lymphocyte indices collectively impact clinical courses. Meanwhile, both CURB-65 (r = 0.31, *p* = 0.001) and NEWS (r = 0.36, *p* < 0.001) show moderate associations.

**Figure 1:**
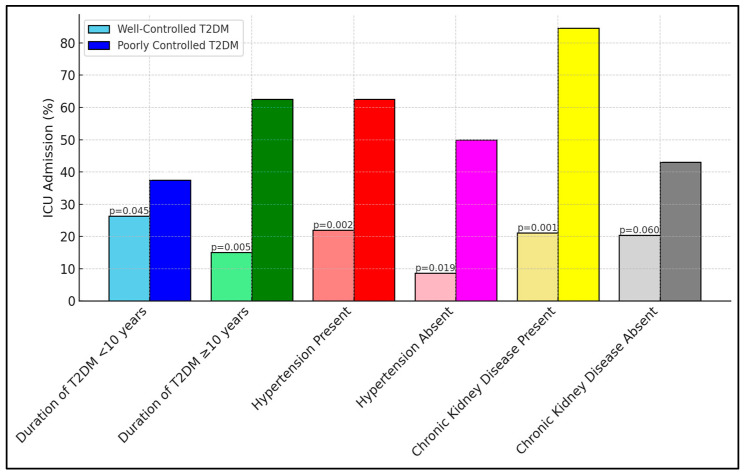
Subgroup analyses for ICU admission.

Table 7 reveals critical insights into ICU admissions among different subgroups, based on the duration of type 2 diabetes mellitus (T2DM) and the presence of comorbid conditions. Notably, the subgroup of patients with poorly controlled diabetes and a longer duration of T2DM (≥10 years) showed a substantially higher rate of ICU admission (62.5%) compared to those with well-controlled diabetes (15.0%), indicating a significant *p*-value of 0.005. Similar trends were observed in patients with hypertension and chronic kidney disease, where poorly controlled diabetic patients exhibited markedly higher ICU admissions, something which was especially striking regarding those with chronic kidney disease (84.6% vs. 21.1%, *p* = 0.001), as presented in Figure 1.

**Figure 2:**
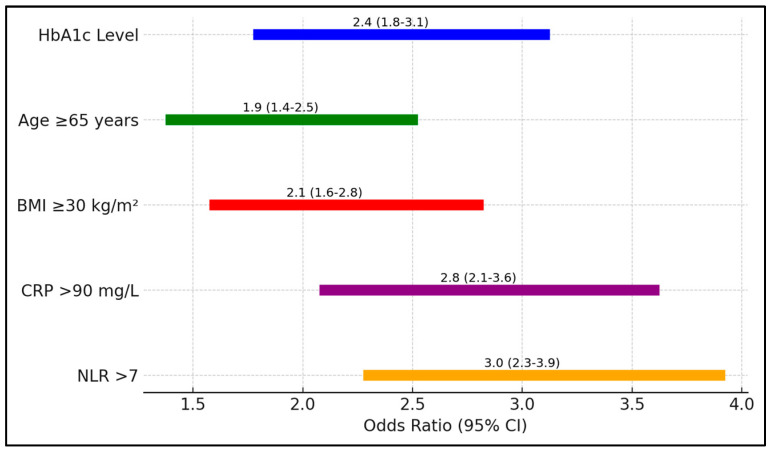
Forest plot analysis of risk factors for ICU admission.

In Table 8, the analysis quantifies the risk associated with various factors for ICU admission among patients with COVID 9 and T2DM. Elevated HbA1c levels posed a 2.4-fold increased risk (95% CI 1.8–3.1, *p* = 0.001), underscoring the critical influence of glycemic control on disease severity. Additional factors such as age ≥ 65 years and a BMI ≥ 30 kg/m^2^ also significantly increased the risk of ICU admission, with odds ratios of 1.9 and 2.1, respectively (Figure 2).

## 4. Discussion

### 4.1. Analysis of Findings

Our findings underscore the pivotal role of glycemic control in dictating the clinical course and inflammatory profiles of T2DM patients hospitalized with COVID-19. Poorly controlled diabetes (HbA1c ≥ 7%) was associated with higher levels of inflammatory markers such as CRP, IL-6, and ferritin, as well as elevated composite indices (NLR, SII). These laboratory data correlate well with the increased severity scores (APACHE II, CURB-65, NEWS) and poorer outcomes, including a higher rate of ICU admission. This suggests that even modest elevations in chronic blood glucose could amplify the immunopathological response to SARS-CoV-2, leading to more severe organ dysfunction.

Our subgroup analyses indicate that advanced age and obesity further compound these effects, aligning with the existing literature that identifies metabolic syndrome as a major risk factor for critical COVID-19 illness. Interestingly, although age ≥ 65 years was distributed similarly across both groups, the poorly controlled cohort consistently showed more severe disease patterns. This discrepancy may be explained by the synergy between chronic hyperglycemia and acute viral inflammation. Furthermore, obesity, which was more prevalent among those with poorly controlled T2DM, might potentiate chronic low-grade inflammation, thereby escalating COVID-19 severity.

The correlations between inflammatory markers and ICU admissions highlight the importance of careful monitoring in clinical practice. For instance, elevated IL-6 and CRP serve as accessible biomarkers that can be measured repeatedly and might prompt early intervention or escalation of care. Severity scores like APACHE II and NEWS complement these laboratory measurements by capturing the multisystem impact of COVID-19, providing clinicians with a multifactorial approach to risk stratification. Taken together, these findings build upon mounting evidence that optimizing glycemic control can mitigate inflammatory surges and may improve the prognosis of T2DM patients during acute viral infections.

In investigating the role of glycemic control on COVID-19 outcomes, two significant studies have presented corroborating evidence on the risks associated with elevated HbA1c levels. Francesco Prattichizzo et al. [25] conducted a systematic review and meta-analysis to explore the impact of pre-admission or at-admission HbA1c levels on COVID-19 mortality among diabetic patients. They found a clear linear relationship between increasing HbA1c levels and the risk of COVID-19 mortality or COVID-19 worsening, with an odds ratio (OR) of 1.01 for HbA1c considered as a continuous variable (*p* < 0.00001), and a more pronounced risk with poor glycemic control (OR 1.15 [1.11, 1.19]; *p* < 0.00001) among various glycemic strata. In a similar manner, the study by Ayaka Yoroidaka et al. [26] also underscored the significance of HbA1c levels in determining COVID-19 severity. They reported that overlooked diabetes was prevalent in 8.7% of COVID-19 inpatients, with higher HbA1c levels being a predictor of severe disease progression (OR 1.45 for each unit increase in HbA1c).

Krishnasamy et al. [27] conducted a multi-center retrospective study, focusing on 1627 adults hospitalized with SARS-CoV-2 pneumonia, finding that elevated HbA1c levels and/or admission hyperglycemia were associated with an increased risk of acute in-hospital cardiovascular events (OR, 1.73; 95% CI, 1.07–2.80), ICU admissions (OR, 1.61; 95% CI, 1.10–2.34), and mortality (OR, 1.77; 95% CI, 1.02–3.07). In a similar manner, the meta-analysis by Vafea et al. [28] reviewed 22 studies involving 11,220 patients, demonstrating that lower HbA1c levels were associated with lower in-hospital mortality (OR, 0.53; 95% CI, 0.37–0.76), particularly evident in diabetic patients with an HbA1c cutoff of 6.5% (OR, 0.34; 95% CI, 0.15–0.77) and 7% (OR, 0.54; 95% CI, 0.32–0.90). Both studies underscore the critical role of glycemic control in influencing the severity and outcome of COVID-19, highlighting the necessity for effective monitoring and management of blood glucose levels in hospitalized patients to mitigate risks and improve prognosis.

Other similar studies delved into the impact of glycated hemoglobin levels on COVID-19 outcomes, presenting important insights but with varying focal points and findings. Alhakak et al. [29] conducted a nationwide study in Denmark, examining 3295 hospitalized COVID-19 patients, and reported that extremely low or high HbA1c levels in patients with diabetes were associated with higher risks of severe infection, ICU admission, and mortality. Specifically, patients with HbA1c levels below 48 mmol/mol or above 64 mmol/mol had notably higher standardized absolute risk differences for adverse outcomes, compared to the reference group with HbA1c levels from 59 to 64 mmol/mol. Similarly, Zheng Zhu et al.’s [30] systematic review and meta-analysis, which included 2577 subjects from nine clinical trials, found that while the relationship between HbA1c as a continuous variable and adverse COVID-19 prognosis was not significant, high HbA1c levels considered as a dichotomous variable significantly increased the risk of mortality (OR, 2.300; 95% CI, 1.679–3.150). Both studies underscore the complexity of HbA1c’s role in COVID-19 prognosis, with Alhakak et al. [29] emphasizing the nonlinear risks associated with varying HbA1c levels, and Zhu et al. [30] confirming the severe risks posed by high HbA1c thresholds, thereby highlighting the need for targeted glycemic control in COVID-19 management strategies.

On the other hand, some studies had different results and observations regarding the effects of serum glucose and HbA1c levels on COVID-19. Barmanray et al. focused on community-acquired pneumonia prior to the COVID-19 pandemic, analyzing data from 38 studies and finding that in-hospital hyperglycemia was significantly associated with increased in-hospital mortality (adjusted OR 1.28, 95% CI 1.09 to 1.50) and ICU admission (crude OR 1.82, 95% CI 1.17 to 2.84), whereas diabetes alone did not show a significant association with these outcomes [31]. Similarly, Kandinata et al. [32] reported that in COVID-19 patients with type 2 diabetes, random blood glucose levels, but not HbA1c, were significantly associated with mortality. This suggests that acute glycemic spikes rather than long-term glycemic control are more predictive of negative outcomes in hospitalized patients, whether for CAP or COVID-19. Both studies underscore the importance of managing acute hyperglycemia over solely focusing on chronic glycemic control, indicating a need for robust glycemic management protocols in hospitals to reduce mortality and severe complications in these patient populations.

Dysregulated diabetes can exacerbate COVID-19 through multiple interrelated mechanisms, creating a hyperinflammatory and immunocompromised state. Hyperglycemia promotes excessive cytokine release, reduces immune cell functionality (such as phagocytosis and T-cell response), and fosters an environment conducive to viral replication and tissue damage [33]. In addition, chronic inflammation and metabolic dysregulation, including elevated levels of advanced glycation end-products and oxidative stress, weaken vascular integrity and organ function, amplifying the likelihood of complications like acute respiratory distress syndrome or multi-organ failure [34]. Consequently, patients with poorly controlled diabetes are at higher risk of severe COVID-19 outcomes, reflecting the synergistic pathophysiological burden of endocrine, metabolic, and immunological dysfunction [35].

While previous research has established that patients with diabetes are at heightened risk for severe COVID-19, our study advances this understanding by specifically examining how varying levels of glycemic control modulate inflammatory markers and clinical severity. By categorizing patients based on HbA1c thresholds, we reveal a clear link between chronic hyperglycemia, more pronounced immune dysregulation, and adverse outcomes. These findings offer novel insights into the mechanistic interplay between long-term glucose management and COVID-19 severity, underscoring the importance of stringent glycemic control in this vulnerable population.

### 4.2. Study Limitations

This study has several limitations. First, its retrospective design may introduce selection biases and limit the ability to establish causation. Although we carefully extracted data and employed standardized definitions, information gaps or inaccuracies in medical records remain possible. Second, the single-center setting constrains generalizability to other populations or regions with different healthcare practices and demographic profiles. Third, we did not evaluate longitudinal changes in glycemic control or inflammatory markers throughout hospitalization. Such serial measurements could provide a more dynamic understanding of the relationships among hyperglycemia, systemic inflammation, and clinical severity. Fourth, we did not account for specific treatments (e.g., antivirals, corticosteroids, and insulin regimens) in our analyses, which could influence both inflammation and outcomes. Moreover, using HbA1c as a dichotomous variable (<7% vs. ≥7%) may oversimplify the relationship between glycemic control and outcomes. Lastly, our sample size, particularly in the subgroups (e.g., patients with obesity), may limit statistical power. Future prospective, multi-center studies are warranted to validate these findings and explore targeted interventions to improve patient outcomes.

## 5. Conclusions

This study demonstrates that among hospitalized COVID-19 patients with type 2 diabetes mellitus, poor glycemic control (HbA1c ≥ 7%) correlates with increased systemic inflammation, greater disease severity, and worse clinical outcomes compared to patients with well-controlled diabetes (HbA1c < 7%). Specifically, higher levels of CRP, IL-6, NLR, and SII in the poorly controlled group reflect a more intense immune activation, which aligns with elevated severity scores such as APACHE II and NEWS. These factors, in turn, translate into higher ICU admission rates and a trend toward increased mortality.

## Figures and Tables

**Table 1:** Baseline demographics.

Variable	Well-Controlled T2DM (*n* = 117)	Poorly Controlled T2DM (*n* = 64)	*p*-Value
Age (years)	62.7 ± 10.6	63.9 ± 9.8	0.4
Male (%)	54.7% (64/117)	59.4% (38/64)	0.5
Duration of T2DM (years)	10.2 ± 4.8	10.4 ± 5.0	0.75
BMI (kg/m^2^)	28.4 ± 4.2	30.2 ± 3.9	0.006
Hypertension (%)	70.1% (82/117)	75.0% (48/64)	0.46
CKD (%)	16.2% (19/117)	20.3% (13/64)	0.46

**Table 2:** Laboratory and inflammatory markers.

Marker	Well-Controlled (*n* = 117)	Poorly Controlled (*n* = 64)	*p*-Value	Mean Diff. [95% CI]
CRP (mg/L)	78.6 ± 23.4	93.4 ± 24.7	0.002	14.8 [6.0, 23.6]
IL-6 (pg/mL)	52.8 ± 15.9	64.2 ± 16.1	0.004	11.4 [4.0, 18.8]
Ferritin (µg/L)	449.2 ± 136.5	524.7 ± 139.2	0.01	75.5 [15.2, 135.8]
Lymphocytes (×10^9^/L)	1.2 ± 0.4	1.0 ± 0.3	0.003	−0.2 [−0.3, −0.1]
NLR	5.7 ± 2.0	7.2 ± 2.3	<0.001	1.5 [0.9, 2.1]
SII (×10^3^)	1039.3 ± 319.5	1344.7 ± 385.6	<0.001	305.4 [148.2, 462.6]

CRP—C-reactive protein; IL-6—interleukin-6; NLR—neutrophil-to-lymphocyte ratio.

**Table 3:** Severity scores at admission.

Score	Well-Controlled (*n* = 117)	Poorly Controlled (*n* = 64)	Mean Diff. [95% CI]	Non-Diabetic COVID Control (*n* = 80)	*p*-Value (Well vs. Poor)	*p*-Value (All 3 Groups)
APACHE II	14.1 ± 4.3	16.8 ± 4.8	2.7 [1.2, 4.2]	13.2 ± 3.9	0.001	0.002
CURB-65	2.1 ± 1.0	2.5 ± 1.1	0.4 [0.1, 0.7]	1.8 ± 0.8	0.03	0.04
NEWS	6.6 ± 2.4	7.7 ± 2.7	1.1 [0.3, 1.9]	5.9 ± 2.2	0.007	0.01

APACHE II—acute physiology and chronic health evaluation II; CURB-65—confusion, urea, respiratory rate, blood pressure, age ≥ 65; NEWS—national early warning score.

**Table 4:** Clinical outcomes.

Outcome	Well-Controlled (*n* = 117)	Poorly Controlled (*n* = 64)	Non-Diabetic Controls (*n* = 80)	Effect Size [95% CI]	*p*-Value (Well vs. Poor)	*p*-Value (All 3 Groups)
ICU Admission (%)	22.2% (26/117)	39.1% (25/64)	15.0% (12/80)	OR 2.24 [1.15, 4.37]	0.02	0.01
Mechanical Ventilation (%)	10.3% (12/117)	18.8% (12/64)	8.8% (7/80)	OR 2.02 [0.84, 4.89]	0.10	0.12
Mortality (%)	6.0% (7/117)	14.1% (9/64)	4.7% (4/80)	OR 2.56 [0.97, 6.77]	0.05	0.03
Length of Stay (days)	12.7 ± 4.2	14.4 ± 4.8	10.9 ± 4.0	1.7 [0.4, 3.0]	0.01	0.01

ICU—Intensive Care Unit.

**Table 5:** Subgroup analysis: age ≥ 65 years and obesity (BMI ≥ 30 kg/m^2^).

Subgroup	Well-Controlled T2DM	Poorly Controlled T2DM	Non-Diabetic Controls (*n* = 80)	Effect Size [95% CI]	*p*-Value (Well vs. Poor)	*p*-Value (All 3 Groups)
Age ≥ 65 years (%)	46.2% (54/117)	48.4% (31/64)	45.0% (36/80)	OR 1.10 [0.65, 1.86]	0.77	0.81
ICU in Age ≥ 65 (%)	29.6% (16/54)	41.9% (13/31)	25.0% (9/36)	OR 1.74 [0.69, 4.41]	0.23	0.26
Obesity (BMI ≥ 30 kg/m^2^)	30.8% (36/117)	48.4% (31/64)	28.8% (23/80)	OR 2.16 [1.14, 4.10]	0.02	0.03
ICU in Obesity (%)	25.0% (9/36)	38.7% (12/31)	17.4% (4/23)	OR 1.90 [0.62, 5.78]	0.24	0.21

**Table 6:** Correlation of inflammatory markers and severity scores with ICU admission.

Variable	Correlation Coefficient (r)	*p*-Value
CRP (mg/L)	0.41	<0.001
IL-6 (pg/mL)	0.45	<0.001
NLR	0.38	<0.001
SII (×10^3^)	0.33	0.001
APACHE II	0.51	<0.001
CURB-65	0.31	0.001
NEWS	0.36	<0.001

**Table 7:** Subgroup analyses for ICU admission based on duration of T2DM and comorbidities.

Subgroup	Well-Controlled T2DM ICU Admission (%)	Poorly Controlled T2DM ICU Admission (%)	*p*-Value
Duration of T2DM <10 years	15/57 (26.3%)	12/32 (37.5%)	0.045
Duration of T2DM ≥ 10 years	9/60 (15.0%)	20/32 (62.5%)	0.005
Hypertension Present	18/82 (22.0%)	30/48 (62.5%)	0.002
Hypertension Absent	3/35 (8.6%)	8/16 (50.0%)	0.019
Chronic Kidney Disease Present	4/19 (21.1%)	11/13 (84.6%)	0.001
Chronic Kidney Disease Absent	20/98 (20.4%)	22/51 (43.1%)	0.06

**Table 8:** Risk assessment for ICU admission.

Risk Factor	Odds Ratio (95% CI)	*p*-Value
HbA1c Level	2.4 (1.8–3.1)	0.001
Age ≥ 65 years	1.9 (1.4–2.5)	0.003
BMI ≥ 30 kg/m^2^	2.1 (1.6–2.8)	0.002
CRP > 90 mg/L	2.8 (2.1–3.6)	0.0005
NLR > 7	3.0 (2.3–3.9)	0.0003

## Data Availability

The data presented in this study are available on request from the corresponding author.
